# Safety and Side Effects of Using Placenta-Derived Decidual Stromal Cells for Graft-versus-Host Disease and Hemorrhagic Cystitis

**DOI:** 10.3389/fimmu.2017.00795

**Published:** 2017-07-11

**Authors:** Arjang Baygan, Wictor Aronsson-Kurttila, Gianluca Moretti, Babylonia Tibert, Göran Dahllöf, Lena Klingspor, Britt Gustafsson, Bita Khoein, Guido Moll, Charlotta Hausmann, Britt-Marie Svahn, Magnus Westgren, Mats Remberger, Behnam Sadeghi, Olle Ringden

**Affiliations:** ^1^Translational Cell Therapy Research Group (TCR), Division of Therapeutic Immunology, Department of LabMed, Karolinska Institutet, Stockholm, Sweden; ^2^Department of Dental Medicine, Karolinska Institutet, Stockholm, Sweden; ^3^Department of Microbiology, Uppsala University Hospital, Uppsala, Sweden; ^4^Department of Pediatrics, Uppsala University Hospital, Uppsala, Sweden; ^5^Charité Universitätsmedizin, Berlin, Germany; ^6^Center for Allogeneic Stem Cell Transplantation, Department of Pathology/Oncology, Karolinska University Hospital, Stockholm, Sweden; ^7^Department of Obstetrics and Gynecology, Karolinska University Hospital, Stockholm, Sweden

**Keywords:** graft-versus-host disease, mesenchymal stromal cells, hematopoietic stem cell transplantation, hemorrhagic cystitis, decidual stromal cells

## Abstract

Mesenchymal stromal cells (MSCs) are increasingly used in regenerate medicine. Placenta-derived decidual stromal cells (DSCs) are a novel therapy for acute graft-versus-host-disease (GVHD) and hemorrhagic cystitis (HC) after allogeneic hematopoietic stem cell transplantation (HSCT). DSCs are more immunosuppressive than MSCs. We assessed adverse events and safety using DSCs among 44 treated patients and 40 controls. The median dose of infused cells was 1.5 (range 0.9–2.9) × 10^6^ DSCs/kg. The patients were given 2 (1–5) doses, with a total of 82 infusions. Monitoring ended 3 months after the last DSC infusion. Three patients had transient reactions during DSC infusion. Laboratory values, hemorrhages, and transfusions were similar in the two groups. The frequency of leukemic relapse (2/2, DSC/controls) and invasive fungal infections (6/6) were the same in the two groups. Causes of death were those seen in HSCT patients: infections (5/3), respiratory failure (1/1), circulatory failure (3/1), thromboembolism (1/0), multiorgan failure (0/1), and GVHD and others (2/7). One-year survival for the DSC patients with GVHD was 67%, which was significantly better than achieved previously at our center. One-year survival was 90% in the DSC-treated HC group. DSC infusions appear safe. Randomized studies are required to prove efficacy.

## Introduction

Acute graft-versus-host disease (GVHD) is a major cause of morbidity and mortality after allogeneic hematopoietic stem cell transplantation (HSCT) (1, 2). Acute GVHD is caused by donor cytotoxic T cell activation against specific recipient histocompatibility antigens (3). There is no specific therapy for severe acute GVHD and the outcome is dismal for severe or steroid refractory disease (4–6).

Hemorrhagic cystitis (HC) is a toxic complication after HSCT that may be caused by the conditioning (7). Mesenchymal stromal cells (MSCs) have a multilineage differentiation and immunomodulatory capacity (8–10). We introduced MSCs as a novel modulatory therapy for acute GVHD and tissue toxicity such as HC after HSCT (11–13). Efficacy of MSC for treatment of acute GVHD was confirmed in several studies (14). The outcome using MSCs for acute GVHD in a long-term follow-up and in partial responders and non-responders was poor (15, 16). MSCs were given to 23 patients with severe acute GVHD between 2001 and 2007 in pilot studies (11, 12, 15, 16). In a randomized double-blind study, 12 patients were given MSCs and 11 were given placebo for acute GVHD between 2007 and 2010 (unpublished data). There was no difference in survival between the two groups. Therefore, it was of interest to investigate immunomodulatory effects of other sources of stromal cells (17, 18).

The placenta protects the fetus from the mother’s immune system during pregnancy and provides a readily available source of stromal cells (19, 20). There is over 100 years of experience of using the placenta to successfully treat burn injuries in Africa (20). These finding and a potentially unlimited supply inspired us to use placental stromal cells for immunosuppression in experimental and clinical trials (20–23). We have isolated stromal cells from the fetal membrane of maternal origin, the so-called decidual stromal cells (DSCs). DSCs inhibit alloreactive T cell proliferation better than other sources of stromal cells (20). Although surface markers are the same, DSCs differ from other sources of MSCs, especially bone marrow (BM-MSC), in several aspects (20, 21). DSCs are half the size of BM-MSCs, they need direct contact to be immunosuppressive *in vitro*, have a better expansion capacity, and seem to tolerate freeze-thawing better (21–24). Like MSCs, third party DSCs are immunosuppressive *in vitro* and *in vivo* (11, 12, 15, 21, 25). DSCs suppressed the production of interferon gamma and interleukin 17 and increased IL-10 in mixed lymphocyte cultures (MLC) as opposed to BM-MSCs that had no effect on these cytokines (20).

BM-MSCs have been proven safe in a large number of studies (26, 27). Because DSCs differ from BM-MSCs, we wanted to evaluate safety and side effects. Like MSCs, DSCs affect coagulation and can stop hemorrhages (23). Because of the procoagulant activities of DSCs and BM-MSCs, thromboembolism was looked for as potential side effects.

## Materials and Methods

### Patients and Ethics

The study included 44 patients, treated from February 2011 to October 2014, with acute GVHD (*n* = 34) or HC (*n* = 10) and 40 controls, 30 with acute GVHD and 10 with HC, treated from August 2003 to May 2014. We obtained ethical approval from Karolinska Institutet to collect stromal cells from placentas after cesarean sections (2009/418-31/4) and to use DSCs clinically for tissue toxicity, hemorrhaging, and grades II–IV acute GVHD after HSCT (2010/452-31/4).

### Preparation of DSCs

After obtaining informed consent, the DSCs were harvested from nine different human term placentas obtained from healthy mothers according to an earlier protocol during elective cesarean-section births (21). Using flow cytometry, the DSCs were positive for CD29, CD44, CD74, CD90, CD105, CD49d, PD-L1, PD-L2, ICAM-1, and HLA-1. They were negative for CD11a, CD14, CD18, CD31, CD34, CD45, CD85, HLA-G, SSEA-3, SSEA-4, CXCR4, VCAM, EpCAM-1, and HLA-2. All DSCs showed normal karyotype. All donors were seronegative for HIV, hepatitis A and B, and syphilis. The DSCs from the various placentas were tested for their capacity to inhibit MLC (20, 21).

### Infusion of Cells

After expansion of the cells to second to fourth passage, the cells were frozen slowly in Dulbecco’s modified eagles medium (Thermo-Fisher Scientific) containing 10% dimethyl sulfoxide (WAK-Chemie Medical GmbH, Steinbach, German7). The cells were thawed at 37°C and washed in CliniMACS PBSEDTA buffer (AmCell Miltenyi Biotec GmbH, Gladbach, Germany) supplemented with either 10% AB-plasma (18 patients) or 5% albumin (CSL Behring GmbH, Marburg, Germany) (25 patients). The cells were resuspended as above at a concentration of 2 × 10^6^ cells/mL. The cell suspension was filtered through a 70-µm cell strainer (BD) before it was transferred to a heparinized syringe. All cell preparations were tested for bacterial contamination before freezing, and from the infusion suspension. DSCs were infused intravenously for 5 min *via* a central venous line. Before and after infusion of DSCs, 5 mL of saline with 12.5–50 IE heparin/mL (depending on weight of the patient) was infused. The patients were checked regularly for 2 h after infusion of the cells. Twenty-one cell infusions were given with cells cultured to passage 2, 31 with cells cultured to passage 3, and 30 with cells cultured to passage 4. The viability of infused cells was median 93% (range 69–99). The dose was 1.5 (0.9–2.9) × 10^6^ viable DSCs/kg. Patients were given median 2 (range 1–5) doses with a total of 82 infusions.

### Patient Characteristics

Patient characteristics are given in Table 1. Because we wanted to be able to compare laboratory values and adverse events, we tried to select patients who had survived at least 3 months. The GVHD controls were matched if possible with the DSC patients for age, donor (sibling versus unrelated), disease, disease stage, donor age, anti-thymocyte globulin (ATG) prophylaxis, donor–recipient sex match, conditioning, cell source, and partly survival after acute GVHD (Table 1). The control group was treated between 2003 and 2014 and was selected among the most recent patients treated for severe acute GVHD and HC. The controls were not treated with DSCs or MSCs. Patients were treated with DSCs for severe acute GVHD, if they did not respond to steroids or they had co-morbidities and were thought not to tolerate long high-dose steroid therapy. If response was not satisfactory, additional weekly doses were given.

**Table 1:** Patient characteristics.

Factor	Decidual stromal cell (DSC)-graft-versus-host disease (GVHD)	DSC-hemorrhagic cystitis (HC)	GVHD-C	HC-C
*N*	34	10	30	10
Age, median (range)	49 (1–68)	43.5 (8–50)	49 (4–63)	35.5 (16–59)
	All DSC patients = 48.5 (1–68)		All controls = 48 (4–63)	
Children (<18 years)	6	1	5	2
Sex (M/F)	20/14	4/6	17/13	10
**Diagnosis:**
Non-malignant	4	1	0	0
Acute leukemia	10	6	8	10
Lymphoma	4	0	4	1
MDS	10	2	7	0
Chronic leukemia	2	1	5	1
Other	4	0	6	0
Disease stage (E/L)	12/22	6/4	6/18	7/5
**Donor:**
MRD	13	2	12	1
MUD	20	5	13	6
MM	1	3	5	5
Donor age, mean (range)	34 (0–68)	41 (0–58)	37 (0–63)	36 (0–55)
Female to male	7	1	6	3
**Conditioning:**				
TBI-based	12	1	7	4
Chemo-based	22	9	23	8
MAC/RIC	12/22	9/1	11/19	9/3
ATG	21	9	20	10
**SC source:**				
BM/PBSCs/CB	9/24/1	2/7/1	2/24/4	1/9/2
TNC dose, mean (range)	7.6 (1.3–24.5)	7.7 (0.4–25)	10.6 (0.3–21.0)	8.1 (0.3–15.6)
CD34 dose, mean (range)	6.5 (0.2–14.2)	6.3 (0.1–9.7)	9.8 (0.1–18.8)	4.9 (0.1–9.7)
**D prophylaxis:**
CsA + MTX	22	9	23	8
Prograf + Sirolimus	11	0	3	2
CsA + Prednisolon	0	1	4	1
HD Cy post SCT	1	0	0	1
G-CSF post hematopoietic stem cell transplantation	7	1	3	7
Bacteremia	10	6	8	8
IFIs	4	2	4	2
**Acute GVHD**				
0	0	4	0	4
I	0	2	0	2
II	13	3	0	5
III–IV	21	1	30	1
Hemorr. cystitis ≥ II	4	10	4	10

*MRD, matched related donor; MUD, matched unrelated donor; MM, mismatched donor; Female to male, number of male recipients with female donors; TBI, total body irradiation; MAC, myeloablative conditioning; RIC, reduced-intensity conditioning; ATG, anti-thymocyte globulin; BM, bone marrow; PBSCs, peripheral blood stem cells; CB, umbilical cord blood stem cells; TNC, total nucleated cell; CSA, cyclosporine; MTX, methotrexate; HD Cy, high-dose cyclophosphamide; G-CSF, granulocyte colony stimulating factor; IFIs, invasive fungal infections; Disease stage, Early (E), first complete remission or first chronic phase, leukemia or non-malignant disease. All other stages were considered late (L); MDS, myelodysplastic syndrome, MDS refractory anemia with ringed sideroblasts, and unspecified MDS with <5% marrow blasts. Late refers to all other disease stages*.

### Conditioning and GVHD Prophylaxis

Myeloablative conditioning before HSCT was cyclophosphamide (120 mg/kg) combined with 12 Gy fractionated total body irradiation (TBI) or oral busulfan (16 mg/kg) (28). Reduced-intensity conditioning consisted of fludarabine (30 mg/kg/day) for 3–5 days combined with busulfan (8 mg/kg), treosulfan (36 mg/kg), and for lymphoma patients, also TBI as previously described (29). GVHD prophylaxis was mainly cyclosporine and four doses of methotrexate (30), or sirolimus and tacrolimus (31, 32). Recipients of hematopoietic cord blood grafts (*n* = 8) were given cyclosporine combined with prednisolone (33).

### Supportive Care

Patients were treated in reversed isolation in hospital, or at home as previously described (34). Infection prophylaxis and other support have already been published in detail (32–36).

### Diagnosis and Treatment of GVHD

Acute GVHD was diagnosed and staged according to the Seattle criteria (37). In patients with diarrhea, colonoscopy and biopsies were performed. Patients with acute GVHD, who did not respond to prednisolone, were given ATG or daclizumab combined with rituximab as second-line treatment. Extracorporeal psoralene and ultraviolet type-A was given as third-line therapy in some patients. Sometimes other immunosuppressive drugs were used if no response was seen, as described previously (35). After the introduction of MSCs, patients who were not responding to prednisolone were initially given MSCs, but more recently they were given DSCs (11, 12, 21).

### Hemorrhagic Cystitis

Patients with HC were treated with Misoprostrol (a prostaglandine analog), given 3 mg three times a day and forced diuresis if required, and they were classified according to published criteria (7). Persistent HC was treated with BM-MSCs or DSCs (13, 38).

### Data Collection

The medical records were read in full, starting from 3 days before DSC infusion and continuing up to 93 days after the last DSC infusion. For the controls, the date of onset of acute GVHD or HC plus 13 days was used as day 0, since 13 days was the median amount of days for DSC-treated patients to receive DSCs after the diagnosis of acute GVHD or HC.

### Laboratory and Vital Data

The parameters analyzed were as follows: blood hemoglobin (g/L), blood leukocytes and neutrophils (counts/L), blood thrombocytes (counts/L), C-reactive peptide (mg/L), aspartate aminotransferase (ASAT; μkat/L), alanine aminotransferase (ALAT; μkat/L), serum albumin (g/L), serum bilirubin (μmol/L), S-potassium (mmol/L), S-creatinine (μmol/L), S-calcium (mmol/L), S-free calcium/ionized calcium (mmol/L), systolic blood pressure (mmHg), and body temperature (°C).

The time intervals chosen for assessment were “before infusion” (0–3 days before infusion), “after infusion” (0–3 days after infusion), “1, 2, 3, and 4 weeks after infusion.”

### Adverse Events

All adverse events from the patient records were noted and then sorted into these categories: acute coronary syndrome, stroke/transient ischemic attack, pulmonary or arterial thrombosis, venous thrombosis, disability, diarrhea, neutropenia, fever, and death. An event of thromboembolic disease or stroke needed to be confirmed using computed tomography or ultrasonography. Disability was divided into five separate categories in order to easily recognize differences between the patient groups: (1) patient requiring oxygenation, (2) muscular cramps, (3) any reduction in mobility, (4) any episode of confusion, hallucinations, or unconsciousness, and (5) sedation. Fever was defined above 38°C and neutropenia as a neutrophil count below 0.5 × 10^9^/L. Mucositis was diagnosed according to WHO criteria (WHO and Book for Reporting Results for Cancer Treatment, Geneva: World Health Organization 1979) (39), patients were categorized only in control and DSC groups for this analysis. The relative frequency of diarrhea, nausea, vomiting, fever, mucositis, and disability in the groups was compared by calculating the proportion of days these symptoms occurred for each patient.

### Infections

Any occurrence of infections was monitored, examining microbiological, radiological, and clinical data from the medical journals at any given time. Virus titers for Herpes Simplex Virus (HSV), adenovirus, Epstein–Barr virus, Varicella-Zoster virus (VZV), and cytomegalovirus (CMV) reactivation were continuously assessed using PCR (40). CMV disease was a symptomatic infection. Invasive fungal infection was diagnosed as either proven or probable (41).

### Statistics

Overall survival was calculated using the Kaplan–Meier method. The Mann–Whitney *U* test was used to compare continuous variables such as age, oral mucositis as well as laboratory, and vital data. Categorical variables were compared using Fisher’s exact test. Three hundred and fourty tests were performed which means that 17 tests were expected to be significant at the 5% level by chance. Four tests may be significant at random at the 1% level. Analyses were performed using the Statistica software (Statsoft, Tulsa, MN, USA).

Frequency of adverse events was compared using descriptive statistics, *p*-values were calculated along with 95% confidence interval using GraphPad Prism software (San Diego, CA, USA). Mean patient age was compared using unpaired two-sided *t*-test. Occurrence of infectious diseases and fungal prophylaxis were compared using Fisher’s exact test.

## Results

### Data Collection and Patient Age

The follow-up time for laboratory values was significantly longer in the DSC-treated group, median 94 days (range 5–235), than in the controls, median 94 (1–94) (*p* = 0.047). There were no statistically significant differences in age, diagnosis, stem cell source, proportion of female donor to male recipients, conditioning, or GVHD prophylaxis between the DSC and the control groups (Table 1).

### Infusion-Related Adverse Reactions

Three patients experienced symptoms associated with infusion of DSCs.

A male transplanted for follicular lymphoma received five DSC infusions for acute GVHD. He experienced fever and chills during the first infusion. He had an ongoing *Staphylococcus epidermidis* sepsis. No negative reactions were observed during the subsequent four DSC infusions that were given 7, 14, 18, and 169 days after the first DSC infusion.

A child was transplanted for pre-B acute lymphoblastic leukemia and received five DSC infusions. During the fourth DSC infusion, the patient experienced headache and dyspnea, and the oxygen saturation dropped to 83%. This was remedied by giving him oxygen support. Before the fifth infusion, he experienced anxiety and was given oxygen support before and during the infusion to counteract this, and the infusion proceeded without any problems.

A male, transplanted for sickle-cell anemia, experienced transient vertigo during the cell infusion. The patients did not experience vertigo thereafter.

### Laboratory Data and Vital Data

There were no significant differences in blood leukocyte counts, C-reactive protein, ASAT, ALAT, creatinine, or bound and free calcium. Hemoglobin, bilirubin, platelet, and albumin levels are presented in Table 2. The controls with GVHD had higher hemoglobin levels than the DSC group at 2 weeks after day 0. Three weeks after day 0, albumin levels were significantly higher in the acute GVHD group treated with DSCs than in the acute GVHD-control group (*p* = 0.03). Bilirubin levels were higher in the GVHD-control group than in the DSC patients, before infusion of DSCs. Platelet counts were not significantly different between any compared groups.

**Table 2:** Hemoglobin, platelet, albumin, and bilirubin levels.

	Before	After	1 week after	2 weeks after	3 weeks after	4 weeks after
	
	Median	Median	Median	Median	Median	Median
**Hemoglobin**						
DSC	103.5	**99.5[Table-fn tfn1]**	100	**104[Table-fn tfn1]**	106	108
CTRL	106	**110[Table-fn tfn1]**	107	**113.5[Table-fn tfn1]**	107.5	103.5
DSC-GVHD	110	101	102	**106[Table-fn tfn1]**	106	109.5
CTRL-GVHD	109	111	109	**114[Table-fn tfn1]**	110.5	102
**Thrombocytes**						
DSC	81	65.5	63.5	59	56.5	68
CTRL	47	52	68	47.5	49	64
DSC-GVHD	**78[Table-fn tfn1]**	**65[Table-fn tfn1]**	**56[Table-fn tfn1]**	**47[Table-fn tfn1]**	**54.5[Table-fn tfn1]**	**61[Table-fn tfn1]**
CTRL-GVHD	**70[Table-fn tfn1]**	**61[Table-fn tfn1]**	**74[Table-fn tfn1]**	**56[Table-fn tfn1]**	**42.5[Table-fn tfn1]**	**64[Table-fn tfn1]**
**Albumin**						
DSC	25	22	26	28	29.5	30
CTRL	28	27	26	28	26	28.5
DSC-GVHD	25	22	26	28	**28.5[Table-fn tfn1]**	29
CTRL-GVHD	27	24	23.5	24	**24[Table-fn tfn1]**	24
**Bilirubin**						
DSC	**9[Table-fn tfn1]**	10.5	10	10	9.5	8
CTRL	**13[Table-fn tfn1]**	14	14.5	12.5	14	13
DSC-GVHD	**9[Table-fn tfn1]**	**12[Table-fn tfn1]**	11	12	10	9.5
CTRL-GVHD	**15.5[Table-fn tfn1]**	**25.5[Table-fn tfn1]**	17	15	18	17

*DSC, decidual stromal cell treated group (*n* = 44); CTRL, controls (*n* = 40); DSC-GVHD, decidual stromal cell treated graft-versus-host disease group (*n* = 34); CTRL-GVHD, graft-versus-host control group (*n* = 30)*.

*Numbers in bold indicate statistical significance using Mann-Whitney U test*.

### Adverse Events, Infections, Relapse of Malignant Disease, and Causes of Death

On day 0, the median WHO mucositis score was 2 (range 0–3) in all DSC patients and 2 (range 0–3) in the controls (*p* = 0.659). There were no significant differences in mucositis score between the groups during the healing phase, to day 12.

Cytomegalovirus reactivation was more common in the controls (*p* = 0.005, Table 3). There was no difference in the frequency of infections, such as pneumonia, septicemia, viral infections, or urinary tract infections. The DSC group had a higher mean prophylactic coverage against invasive fungal infections (IFIs) (Table 3).

**Table 3:** Significant findings in frequency of cytomegalovirus (CMV) reactivation, non-invasive fungal infection, and mean prophylactic coverage.

	DSC	CTRL	DSC-GVH	CTRL-GVH	DSC-HC	CTRL-HC
**CMV-PCR positivity**						
Proportion	0.749	0.698	0.792	0.683	0.612	0.725
*N* (Serum assays)	506	311	393	218	125	109
*p*-Value	0.123		**0.005**		0.093	
**Non-invasive fungal infections**						
*N* (patients)	6	14	5	10	1	4
*p*-Value	**0.039**		0.1379		0.303	
**Posaconazole prophylaxis**						
Proportion	0.516	0.273	0.551	0.303	0.389	0.194
*N* (days)	4,128	2,814	3,285	2,017	890	797
*p*-Value	**<0.0001**		**<0.0001**		**<0.0001**	
***P. jirovecii prophylaxis***						
Proportion	0.853	0.865	0.842	0.822	0.896	0.971
*N* (days)	4,128	2,854	3,285	2,187	890	837
*p*-Value	0.163		0.057		**<0.0001**	

*Numbers in bold indicate statistical significance. DSC, decidual stromal cell treated group (*n* = 44); DSC-GVHD, decidual stromal cell treated graft-versus-host disease group (*n* = 34); DSC-HC, decidual stromal cell treated hemorrhagic cystitis group (*n* = 10); CTRL, controls (*n* = 40); CTRL-GVHD, graft-versus-host control group (*n* = 30); CTRL-HC, hemorrhagic cystitis control group (*n* = 10). “Difference” indicates the 95% CI of the difference between the groups, *p*-value calculated using two-sided *t*-test. The “proportion” value indicates the proportion of positive serum assays for CMV-PCR positivity and the proportion of days with prophylactic therapy. *p*-Value calculated using Fisher’s exact test*.

Invasive fungal infection was observed in six of the DSC patients and six of the controls (Table 4). In the DSC group, there were three probable *Aspergillus* infections, one probable and two proven zygomyzetes infections (one *Rhizopus microsporus* and one *Rhizopus arrhizus*). Among the controls were two probable *Aspergillus* infections, one probable and one proven mold infection, and two probable zygomyzetes infections. One of the latter later grew out *Lichtheimia corymbifera*.

**Table 4:** Frequency of relapse, invasive fungal infection, and causes of death.

	DSC	CTRL	DSC-GVHD	CTRL-GVHD	DSC-HC	CTRL-HC
**Relapse**						
	2	2	2	0	0	2
*p*-Value	1.000		0.474		0.474	
**Invasive fungal infections**						
	6	6	6	4	0	2
*p*-Value						
**Cause of death during observational period (***n***)**						
*Infection*	5	3	4	2	1	1
*p*-Value	0.467		0.676		1.000	
*Other/GVHD*	2	7	2	6	0	1
*p*-Value	0.079		0.139		1.000	
*Respiratory failure*	1	1	1	1	0	0
*p*-Value	1.000		1.000		1.000	
*Circulatory failure*	3	1	3	1	0	0
*p*-Value	0.618		0.616		1.000	
*Thromboembolism*	1^a^	0	1	0	0	0
*p*-Value	1.000		1.000		1.000	
*Multiple organ failure*	0	1	0	1	0	0
*p*-Value	0.476		0.469		1.000	

*Numbers indicate number of patients. Numbers in bold indicate statistical significance. DSC, decidual stromal cell treated group (*n* = 44); DSC-GVHD, decidual stromal cell treated graft-versus-host disease group (*n* = 34); DSC-HC, decidual stromal cell treated hemorrhagic cystitis group (*n* = 10); CTRL, controls (*n* = 40); CTRL-GVHD, graft-versus-host control group (*n* = 30); CTRL-HC, hemorrhagic cystitis control group (*n* = 10)*.

*^a^In this case, the thromboembolism was a brain infarction*.

Acute GVHD/HC DSC-treated patients had the following events: HSV reactivation; 8/3, CMV reactivation; 22/6, CMV disease; 5/3, VZV reactivation; 8/3, posttransplant lymphoproliferative disorder; 0/0, IFIs 6/0, septicemia; 10/3, pneumonia; 6/1, and leukemic relapse; 2/0.

There were no significant differences between the DSC group and the controls concerning leukemic relapse or cause of death (Table 4).

### Thromboembolism, Stroke, and Transient Ischemic Attack

Four patients in the DSC-treated group and two in the control group had an adverse event with thromboembolism, stroke, or transient ischemic attack during the observation period (*p* = 0.678). Two DSC-treated patients and one control had had thromboembolic disease in the same location before the observation period. The two remaining DSC patients had IFIs.

A female patient transplanted for chronic myelomonocytic leukemia received two DSC doses for acute GVHD. A CT scan of the head and brain showed a suspected bleeding in the basal frontal region of the brain 45 days after the second DSC infusion. She had probable *Aspergillus* infections. The patient was alive during the 155 days observational period, but died later.

A female patient transplanted for pre-B acute lymphoblastic leukemia received four DSC doses for acute GVHD. The patient had a CT diagnosed subarachnoid bleeding and died 5 days after the fourth DSC infusion. She had proven rhizopus fungal pneumonia. The patient had an observational period of 115 days with posaconazole prophylaxis during 93 days.

A male control patient transplanted for metastatic renal cell carcinoma had a thrombosis in the right popliteal vein on the 65th day of observation.

### Hemorrhage and Transfusions

Hemorrhaging other than HC was seen in 36% of all DSC patients and in 40% of all controls (*p* = 0.82). The DSC patients requiring transfusions received a median of 9 (range 1–130) erythrocyte transfusions, as opposed to 8 (range 2–28) for the controls (*p* = 0.14). The corresponding figures for platelet transfusions in the two groups were 9 (range 1–74) and 9 (range 2–47), respectively (*p* = 0.23).

### Survival

**Figure 1:**
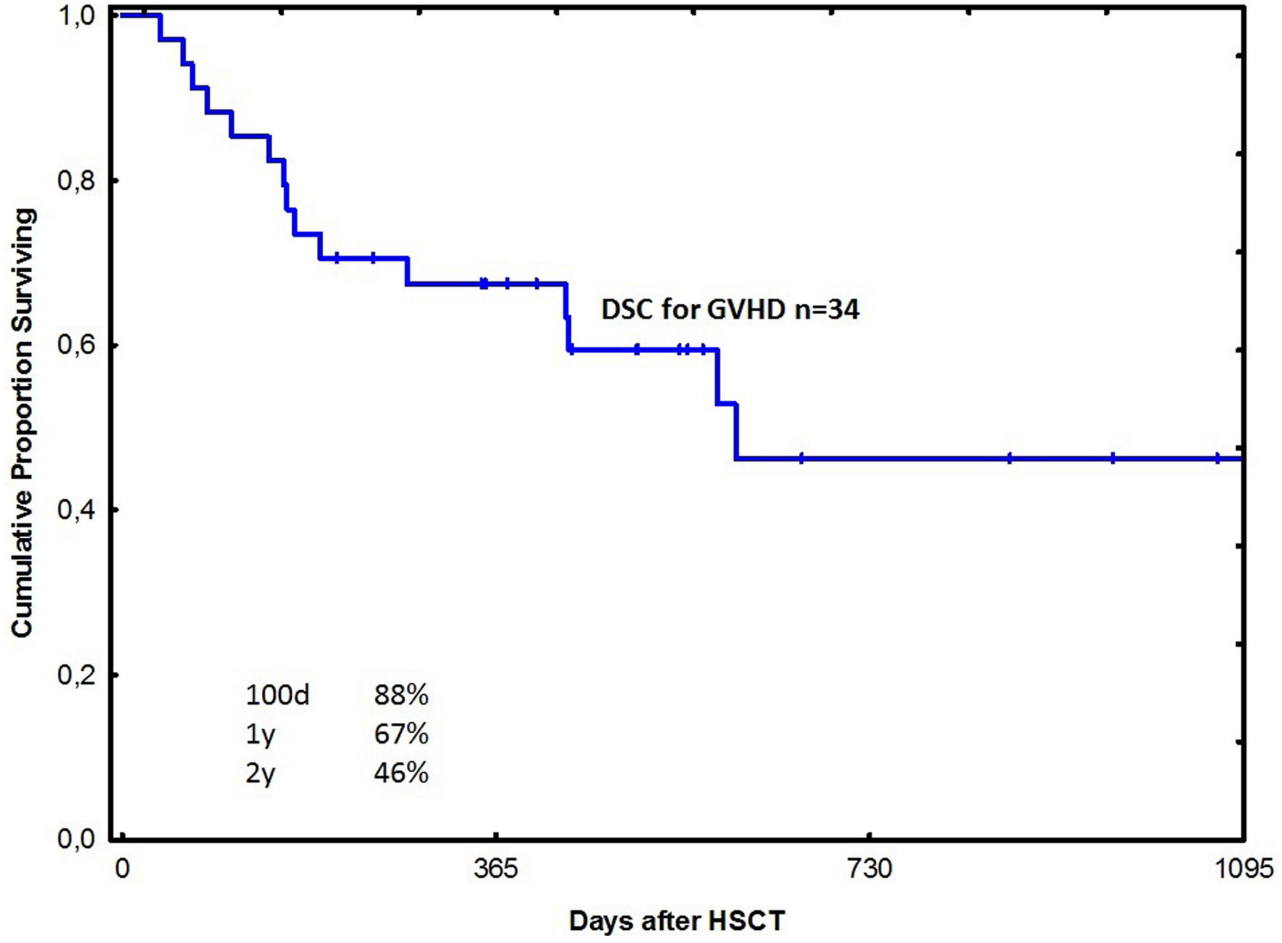
Probability of survival among patients with acute graft-versus-host disease (GVHD) treated with decidual stromal cells (DSCs).

**Figure 2:**
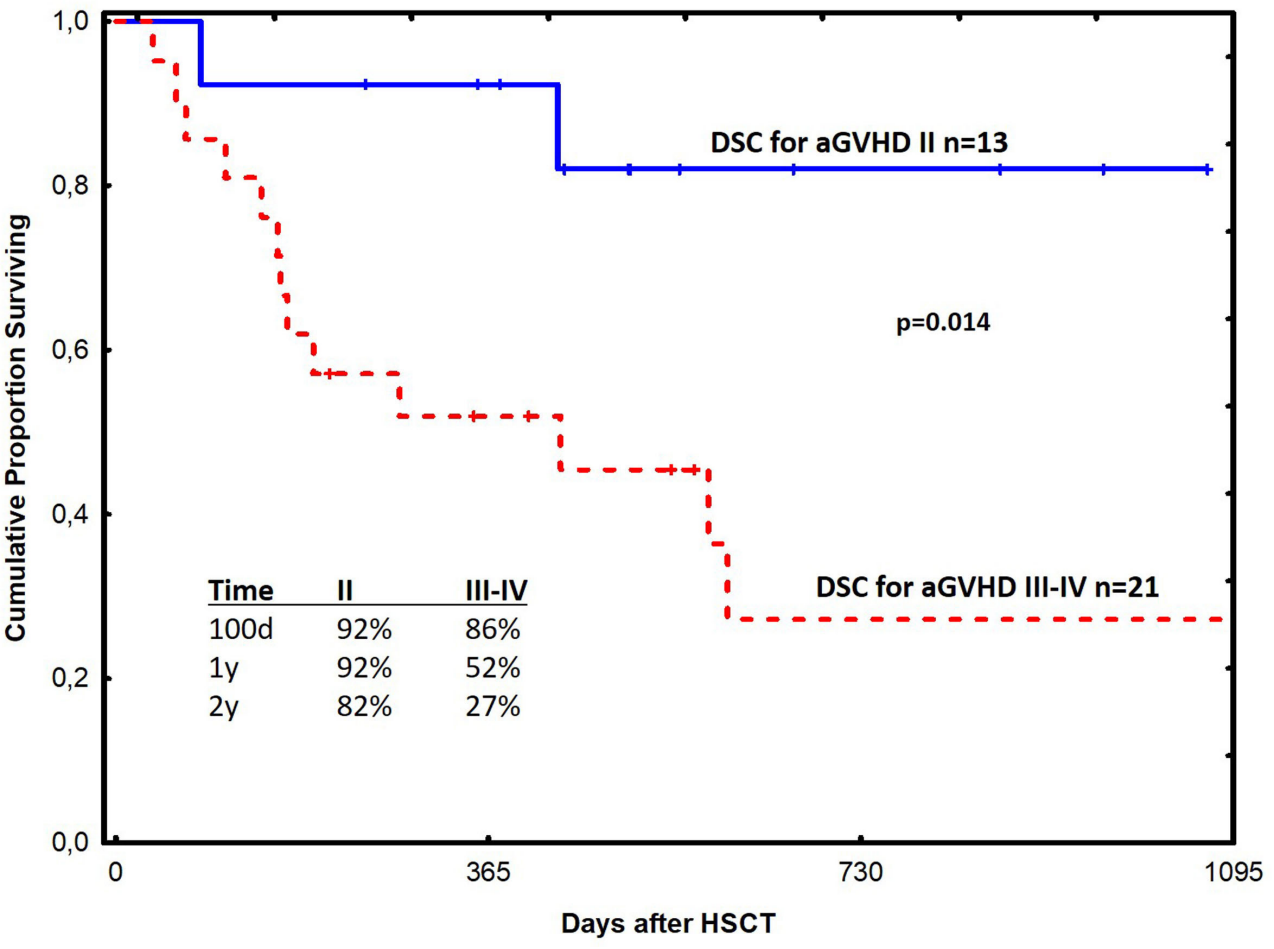
Probability of survival among patients with acute graft-versus-host disease grade II and III–IV respectively (GVHD) treated with decidual stromal cells (DSCs).

**Figure 3:**
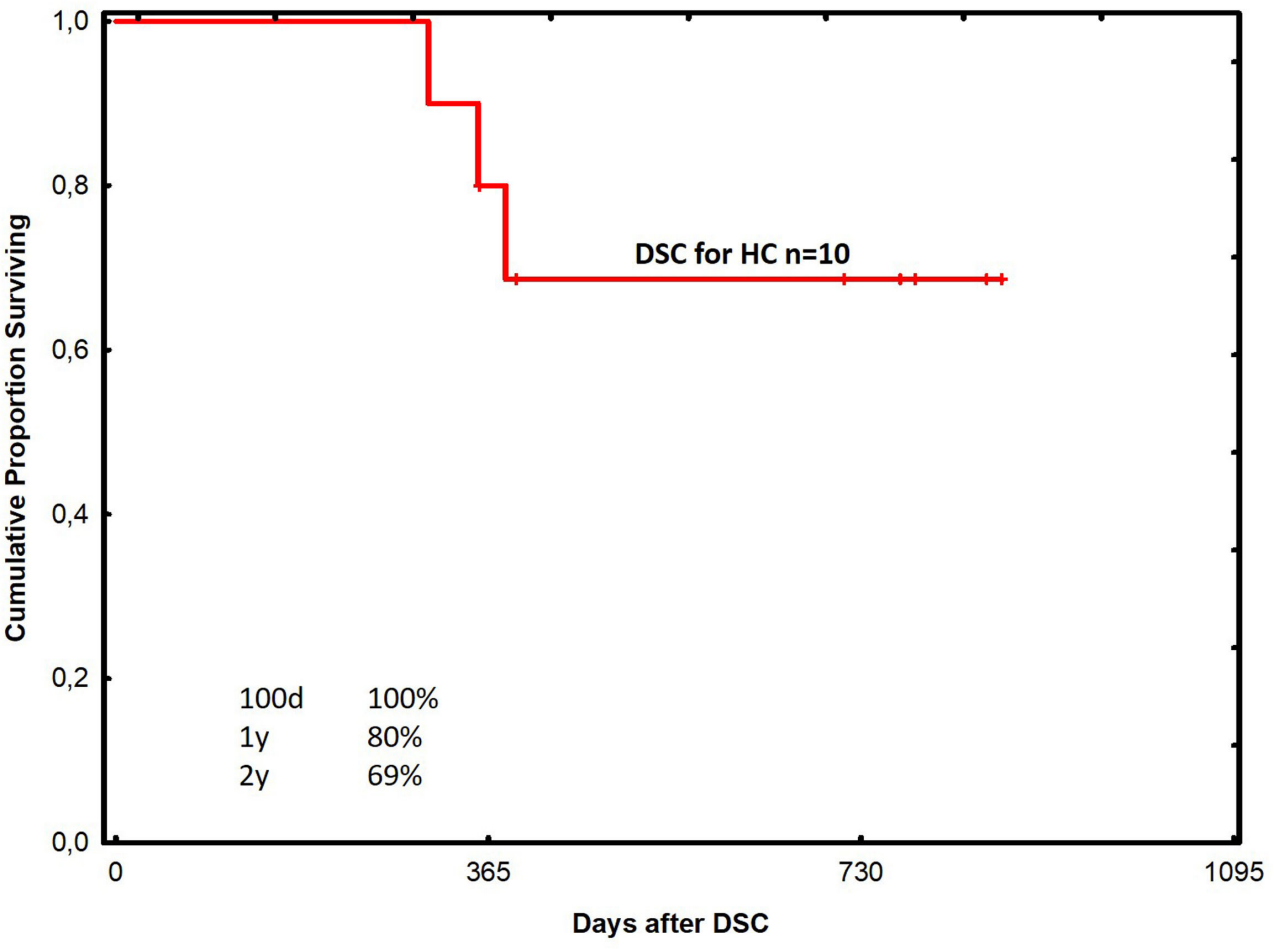
Probability of survival among patients with hemorrhagic cystitis treated with decidual stromal cells.

The probability of survival at 1 year was 67% in all acute GVHD patients treated with DSCs (Figure 1). Among patients with grade II and III–IV acute GVHD, the corresponding figures were 92 and 52% in the two groups, respectively (Figure 2). One-year survival was 90% in the HC patients treated with DSCs (Figure 3).

## Discussion

The data from this study suggest that DSCs are safe to use in the GVHD and HC settings, at the currently applied cell dose of 1–3 × 10^6^ DSCs/kg using low-dose heparin at infusion (21). The adverse events seen in these patients with severe acute GVHD and HSCT are commonly seen in patients with severe acute GVHD. Systolic blood pressure, body temperature, and serum albumin levels were higher at some time intervals in the DSC-treated patients compared to the controls, but they were never above normal range. There were no significant differences in severe adverse events or causes of death between the two groups. The causes of death were those usually seen in patients with GVHD or HC after HSCT (1, 2, 4–6).

With longer follow-up, some problems with BM-MSC treatment have become apparent, such as increased risk of pneumonia (36), invasive fungal infection (16), posttransplant lymphoproliferative disease (42), and a decrease in immune reconstitution (43). However, many more patients will be required to find out whether or not any specific complication may be increased by treatment with DSCs. Serum albumin levels have been shown to be predictive of survival in patients with acute gastrointestinal GVHD (44) and the higher albumin levels seen in the DSC patients with GVHD may be a positive effect of DSC treatment.

In a previous study with a total of 99 BM-MSC infusions, two episodes of angina pectoris were reported in one patient (27). DSC infusion-related adverse reactions, including vertigo and reduced oxygen saturation, seemed to be closely associated with DSCs infusion. The patient who developed fever and chills during DSC infusion had an ongoing septicemia, so a role of DSC infusion in this case is less likely. A meta-analysis, including 1,012 patients treated with MSCs, also showed that stromal cells are safe to infuse (26). In that study, there was a significant correlation between MSC infusion and transient fever. A slightly elevated fever was also observed in the DSC patients, compared to the controls.

Mesenchymal stromal cells and DSCs have the capacity to modulate the coagulation system and have, thus, been used to treat hemorrhaging (13, 38, 45). Even at early passages, DSCs express significantly higher levels of tissue factor than MSCs and, thus, warrant special preparation with low-dose heparin to minimize the potential risk for thromboembolism (45). Because DSCs and MSCs first home to the lung (46), pulmonary embolism should be looked for. The adverse reaction with reduced oxygen saturation during DSC infusion in one case, may be due to congestion with DSCs in the lung, rather than embolism formation. We did not see an increase in pulmonary embolism after DSC infusion. Pulmonary embolism has been reported after infusion with adipose-derived MSCs (47). Similarly, Acosta et al. documented two cases of peripheral microthrombosis upon application of adipose MSCs in two diabetic patients, associated with altered fibrinolytic activity (48). This was accompanied by increased levels of fibrinolysis marker D-dimer in venous blood. Elevated levels of D-dimer were also monitored by Stephenne et al., after systemic infusion of human adult liver progenitor cells (49), which could be antagonized by anti-thrombin therapy. We also found transiently increased levels of D-dimer shortly after infusion of DSCs with low-dose heparin, which rapidly dropped back to baseline levels, suggesting only minimal activation of innate immune cascades (45).

Anticipated adverse events after any immunosuppressive therapy, including DSCs, are increased risks of infections due to hampering of the immune recovery after HSCT. There was no difference in the frequency of IFIs between the groups. This may support the idea that DSCs do not increase the risk of IFIs. It should be noted that the DSC group had a different fungal prophylaxis coverage. In the earlier patients, ketoconazole or no fungal prophylaxis was used. In the more recent patients, posaconazole was given since this had recently become available. Further studies are needed to find out if this prophylaxis has been effective or not. There is a report that MSCs gave a high incidence of IFIs after HSCT (16). Whether or not stromal cells affect immunity to fungi is now being investigated in mouse and pig models.

Cytomegalovirus reactivation was higher in the GVHD-control group than in the DSC group. This difference was significant (*p* = 0.005) and may be important. It is possible that pharmacological immunosuppression given to the controls affected immunity to CMV much more than DSC therapy. This is supported by experimental data, which show that MSCs have not suppressed CMV-cytotoxic T-cells, while they suppress alloreactive T-cells (50).

It is important to compare the results using DSCs with those using other immunosuppressive treatments for acute GVHD. Both groups were treated with cyclosporine and high doses of corticosteroids. The controls were also treated with a wide range of immunosuppressive drugs.

Safety was the objective of this study. Anyhow, survival in the GVHD and the HC groups seem promising using DSC, when compared to previous experience using other therapies at our unit (12, 18, 51). Patients with grade II acute GVHD had a better survival probably because early therapy is of importance for outcome. It is also possible that some of these patients may have recovered with conventional therapy. One-year survival of 52% in the grade III–IV acute GVHD group is also promising. This is much better than what is reported in the literature (4–6).

There are some obvious advantages of using DSCs as opposed to other sources of stromal cells. There is an unlimited supply of placentas. The expansion rate is much higher (21), immunosuppressive capabilities may be higher because *in vitro* in mixed lymphocyte culture DSC inhibition was more consistent than using BM-MSCs (20). Bone marrow aspiration is somewhat painful for the donor. For anti-inflammatory purposes, there is no need for differentiation to cartilage and bone which is one of the features of BM-MSCs (22). DSCs are only half the size of BM-MSCs so the risk of trapping in capillaries may be lower (23). One purpose of the placenta is to protect the fetus from the mother’s HLA-incompatible immune system. Therefore, immunosuppression is a major task for placental stromal cells.

The limitations of this study are obvious, it is not randomized, historic controls were used and the study was retrospective. Therefore, the data have to be taken with caution. We wanted to include controls to show which side effects are commonly seen in HSCT patients regardless of posttransplant therapy for severe acute GVHD. So far, we have not seen an increase of side effects using DSCs. There were only three adverse infusion reactions, and they were transient. The rates of malignant relapse and causes of death were similar in the DSC group and the controls. It should be noted that the follow-up for leukemic relapse was short. However, with more patients and longer follow-up, infections, leukemia relapse, chronic GVHD, and secondary malignancies, and other late problems after MSCT, need to be reevaluated.

Future directions include planning of randomized multicenter studies using DSCs compared to placebo for acute GVHD and HC, respectively, with early response as a primary endpoint. We also have ethical approval to explore DSCs for other inflammatory disorders such as inflammatory bowel disease, acute necrotizing pancreatitis, autoimmune inflammatory neurological disorders, and acute respiratory distress syndrome (ARDS). A patient with ARDS following HSCT was successfully cured by DSCs (52).

In conclusion, the data from this study, as a whole, do not indicate that DSC infusions would have a negative physiological effect on the patients, compared to other second- or third-line immunosuppressive drugs used for severe acute GVHD. DSCs also appeared to be safe in the HC patients. This study serves as a basis for future controlled, randomized trials to evaluate the efficacy of this novel therapy for acute GVHD and HC.

## Ethics Statement

All subjects gave written informed consent in accordance with the Declaration of Helsinki and in the case of minors written consent was given by their legal guardians. We obtained ethical approval from Regionala etikprövningsnämnden i Stockholm to collect stromal cells from placentas after caesarean sections (2009/418-31/4), and to use DSCs clinically for tissue toxicity, hemorrhaging, and grades II–IV acute GVHD after HSCT (2010/452-31/4).

## Author Contributions

AB performed the data collection, categorical, and descriptive statistics. WA-K, GM, BT, BK, CH, and B-MS performed data collection. GD contributed to analysis of oral mucositis. LK performed the review of invasive fungal infections. BG and MW collected elective cesarean sections. MR performed statistical analysis on laboratory parameters. Everyone contributed to the writing.

## Conflict of Interest Statement

The authors declare that the research was conducted in the absence of any commercial or financial relationships that could be construed as a potential conflict of interest.
